# Testing the Effectiveness of the Health Belief Model in Predicting Preventive Behavior During the COVID-19 Pandemic: The Case of Romania and Italy

**DOI:** 10.3389/fpsyg.2021.627575

**Published:** 2022-01-12

**Authors:** Johannes Alfons Karl, Ronald Fischer, Elena Druică, Fabio Musso, Anastasia Stan

**Affiliations:** ^1^School of Psychology, Victoria University of Wellington, Wellington, New Zealand; ^2^D’Or Institute for Research and Education, São Paulo, Brazil; ^3^Centre for Applied Behavioral Economics, Department of Applied Economics and Quantitative Analysis, University of Bucharest, Bucharest, Romania; ^4^Department of Economics, Society and Politics, University of Urbino “Carlo Bo”, Urbino, Italy

**Keywords:** Health Belief Model, COVID-19, preventive behavior, lockdown, culture, health behavior, measurement invariance

## Abstract

We use a cultural psychology approach to examine the relevance of the Health Belief Model (HBM) for predicting a variety of behaviors that had been recommended by health officials during the initial stages of the COVID-19 lockdown for containing the spread of the virus and not overburdening the health system in Europe. Our study is grounded in the assumption that health behavior is activated based on locally relevant perceptions of threats, susceptibility and benefits in engaging in protective behavior, which requires careful attention to how these perceptions might be structured and activated. We assess the validity of the HBM in two European countries that have been relatively understudied, using simultaneous measurements during acute periods of infection in Romania and Italy. An online questionnaire provided a total of (*N* = 1863) valid answers from both countries. First, to understand individual difference patterns within and across populations, we fit a General Linear Model in which endorsement was predicted by behavior, country, their interaction, and a random effect for participants. Second, we assess the effect of demographics and health beliefs on prevention behaviors by fitting a multi-group path model across countries, in which each behavior was predicted by the observed health belief variables and demographics. Health beliefs showed stronger relationships with the recommended behaviors than demographics. Confirming previously reported relationships, self-efficacy, perceived severity, and perceived benefits were consistently related to the greater adoption of individual behaviors, whereas greater perceived barriers were related to lower adoption of health behaviors. However, we also point to important location specific effects that suggest that local norms shape protective behavior in highly contextualized ways.

## Introduction

The interest in psychological theories able to contribute to a design of effective public health interventions and health promotions is high (Murphy and Bennett, 2004; Uutela et al., 2004). This is particularly true in the current environment where public health officials need insights into effective COVID – 19 responses (Bavel et al., 2020), which has severely impacted many aspects of individuals lives across the globe (Osei-Tutu et al., 2021b). At the same time, there is increasing evidence that protective behaviors are culturally molded, requiring a focused examination of perceptions and behaviors within their respective contexts (Fischer and Karl, 2021). We focus on one of the most successful frameworks in the literature, the Health Belief Model (HBM) (Rosenstock, 1974b) and use a cultural psychology perspective (Fontaine, 2011; Wang, 2016) to examine how individuals in two European contexts perceive core constructs within the theory and how well this model works for COVID-19 relevant health behaviors across two cultural contexts. Cultural psychology focus on the interplay between the person, the mind and culture (Shweder, 1991) and tries to understand how beliefs and behaviors are interrelated within cultures. Cultural psychology permits careful comparisons, but focuses on processes (how are beliefs related to behaviors) rather than a variable focus in cross-cultural psychology which explicitly focuses on quantitative comparison. A further distinction is that classic cross-cultural psychology assumes that culture is an external variable that can be easily measured with self-report measures and be treated as an antecedent, cultural psychology does not assume that cultural processes are distinct and conceptual antecedents that need to be measured separately, but rather form part of all measures (e.g., Greenfield, 2000; Smith et al., 2013). Hence, we use this cultural perspective to examine how a model of beliefs relates to individual behaviors during the early stages of the COVID-19 pandemic.

The HBM proved effective in the past in describing a wide range of preventive behaviors for diseases and behaviors that are well documented, increase the probability of early detection of diseases and for which implications of any behavior changes are generally well understood (Carpenter, 2010; Sulat et al., 2018). However, in most cases the contexts where the model has been applied and tested were relatively established health contexts, which allowed people to understand and assess risks to make informed decisions on their personal health behavior (Chen and Land, 1986; Bond et al., 1992; Ahmadi Jouybari et al., 2017; Fall et al., 2018; Jeihooni et al., 2019; Khani-jeihooni et al., 2020). Importantly, any behavior is culturally shaped, especially if behavior affects others and individuals strategically adapt their responses to align with expectations of others (Yamagishi et al., 2008). This cultural interpretation of behavior is immediately relevant for the HBM because the target of the behavior is crucial. Previous research primarily focused on preventive behaviors related to non-communicable diseases or conditions, which are typically individually focused behaviors that differ to a great extent from those related to pandemics where the actions of each individual have follow-on effects on others. Some cultural environments are more likely to focus the attention of individuals toward their group members, in particular cultural environments emphasizing interdependence (Markus and Kitayama, 1991). To the extent that individuals are culturally conditioned to be concerned about the wellbeing of others, their behavior in a pandemic environment is likely to change. At the same time, even within more independent and individualistic contexts, health interventions have much to gain by emphasizing the wellbeing of others, as the case study of a highly individualistic country such as New Zealand has demonstrated (Manning, 2021).

Our first goal is therefore to explore whether the HBM can be applied in such an acute pandemic context that has collective action properties (Fontaine, 2011; Templeton et al., 2020; Fischer and Karl, 2021). To the best of our knowledge, there is relatively little work that takes a cultural psychology perspective to examine how perceptions within the HBM operate within and across cultural contexts. In addition, insufficient evidence regarding the effectiveness of the HBM model in predicting the adoption of recommended behaviors in emergency or high-risk situations that vary across contexts and affect a large number of individuals and are marked by high levels of anxiety. As mentioned previously, the relatively limited literature available suggests that the HBM seems to work better in North America and Western Europe when the targeted behavior is focused on prevention of individually relevant risk factors, compared to adherence to recommended behaviors during an acute public crisis (Carpenter, 2010; Sulat et al., 2018). This better alignment of individualistically focused behaviors in more individualistic oriented contexts could be expected from a cultural perspective (Smith et al., 2013, for divergence of promotion vs. prevention focused messages in United States and British contexts vs. Japan and other East Asian countries, see Hamamura et al., 2009, Uskul et al., 2009). This makes the COVID – 19 pandemic a unique and valuable context to test the applicability of the overall framework. Given the absence of effective medical treatment or vaccines against COVID – 19 at the outset of the pandemic as well as the rapid spread of the virus, the only effective protection and prevention measures available were behavior based. Even today with the widespread availability of vaccines, the most effective interventions are behavior-based interventions and they remain important with the emergence of new variants (Bish and Michie, 2010; Park et al., 2010; Agüero et al., 2011; Fischhoff et al., 2018). However, these preventive behaviors recommended by local and national governments depend on the cooperation of the population which can substantially vary across cultural contexts (Ai et al., 2021). Even with the availability of vaccines, governments depend on their citizens to cooperate in vaccine uptake and to follow continuing health guidelines till the pandemic is under control. Here, cultural perspectives are important as behavior is typically strategic and follows situational logics (Yamagishi et al., 2008; Chiu et al., 2010). Hence, it is crucial to study which variables may influence adherence to official health guidelines, and whether pre-existing theoretical backgrounds can facilitate the adoption of these guidelines.

Second, although there has been support for the overall model in general in a number of different cultural contexts, there is very limited research on the relevance of these perceptions and the comparative effectiveness of the HBM in different social, economic and cultural contexts. Our second goal is to directly test the validity of the HBM for predicting a variety of behaviors that had been recommended during the initial stages of the pandemic for containing the spread of the virus and to prevent overburdening the health system during the first COVID – 19 lockdowns, in two European countries, Romania and Italy. As a secondary goal, we also examine whether individuals in these two contexts perceive the core constructs in the same way, as it is well established that culture and mind reciprocally constitute each other (Kim, 2000; Shweder, 2000). Therefore, we add to the existing research by explicitly exploring the performance of the model in predicting preventive behavior within specific cultural contexts. We include two countries that are located in close geographic proximity, share closely related languages but have different profiles of infection susceptibility and severity at the time of measurement. These two countries differ principally along survival vs. self-expression values (Welzel, 2013), which are important for health behaviors and the control of infectious diseases (Schaller, 2011). Therefore, we can rule out a number of competing explanations linked to shared social and cultural aspects due to a common Latin heritage, and examine the extent to which the HBM is dependent on the interaction between cultural values related to protection vs. self-expression values and the state of the health system. Taking this cultural psychology perspective, we offer new insights into the role of cultural context at different stages of dissemination of the virus and on broader dynamics of adopting health behavior during a global pandemic.

Finally, an important part of any cultural psychology analysis is to provide a better understanding of individual behavior in context. Hence, we assess to what extent different demographic groups within each culture adopted the recommended preventive behaviors, adoption further referred to as adherence. This adds new evidence on individual strategies at a behavioral level and can help health officials in identifying groups that may need specific targeting for reducing risk behaviors within their cultural context.

In summary, our contributions are threefold: (a) report an application of the HBM in an acute crisis setting, (b) explicitly test the cultural validity of the model in two closely related cultural contexts that vary in (1) the level of infection rates and (2) salient socio-economic characteristics such as income rates, health infrastructure and (3) in survival vs. self-expression values which are important cultural orientations that are relevant for reducing infections. Finally, (c) we explore demographic differences to provide insights into the behavior of individuals within cultural contexts.

The rest of the paper is organized as follows: the next section presents the HBM and the cultural context as well as pandemic situation in Romania and Italy when the data was collected; Section “Materials and Methods” provides information about data, measurement and methods; Section “Results” presents the results, while the final sections present the findings, discuss the limitations as well as the theoretical and practical implications of our work.

## Theoretical and Practical Background

### The Health Beliefs Model

The Health Beliefs Model traditionally includes four major types of beliefs: Perceived susceptibility, perceived severity, perceived benefits of preventive actions, and perceived barriers (Rosenstock, 1974a,b). The belief to be able to successfully adopt the behavior, also known as self – efficacy, was added later (Rosenstock et al., 1988), and has been shown to improve the applicability of the model (Champion and Skinner, 2008). Previous studies suggested that barriers and benefits are the strongest predictors of health behavior (Carpenter, 2010; Sulat et al., 2018), with stronger effects for these two variables when focusing on prevention behaviors compared to acute diseases/sickness.

The HBM has been shown relevant for influenza vaccinations, breast self-examination, diet, exercise, smoking and seat-belt use (Prentice-Dunn and Rogers, 1986), HIV (Steers et al., 1996), Type 2 Diabetes Mellitus (Tan, 2004; Chao et al., 2005), dental health (Chen and Land, 1986), adherence to disease modified therapy in multiple sclerosis (Turner et al., 2007; Yoshitake et al., 2019), skin cancer (Jeihooni and Rakhshani, 2019), oral cancer (Jeihooni et al., 2019), nutritional behaviors (Vahedian-Shahroodi et al., 2019), or developing preventive behaviors in young adults (Luquis and Kensinger, 2019).

There is relatively little work on the HBM from a cultural psychology perspective (Arnault, 2018). Self-efficacy is one core component of HBM and conceptualizations of self-efficacy have been shown to systematically vary by cultural models of self-hood (Markus and Kitayama, 1991; Oettingen, 1995; Vignoles et al., 2016). Similarly, the literature regarding the effectiveness of the model in contexts of epidemics, including virus outbreaks, is scant. We found research addressing preventive behavior based on the HBM paradigm in case of seasonal influenza (Karimi et al., 2016; Ahmadi Jouybari et al., 2017; Fall et al., 2018), and the H1N1 influenza (Rezaeipandari et al., 2018; Zhang et al., 2019; Khani-jeihooni et al., 2020). These studies found that the HBM framework is effective in predicting preventive behavior in case of seasonal influenza, however, the predictive power of the HBM dimensions differs by context. In Iran, the most influential predictors of preventive behavior in case of influenza were perceived susceptibility and severity, along with self-efficacy (Ahmadi Jouybari et al., 2017), in France the best predictor was self-efficacy (Fall et al., 2018), whereas in Canada perceived susceptibility, benefits and barriers were all strongly correlated with health behavior (Karimi et al., 2016). However, each of these studies was conducted in isolation and it is not possible to determine whether the individual components were perceived in similar ways by participants (Fischer and Karl, 2019). Therefore, there is relatively little literature available that provides insights whether the perceptions of core concepts with the HBM are perceived similarly or not within distinct cultural contexts.

The context of COVID-19 requires evidence-based practices to provide more effective protection of the most vulnerable within a population. The importance of health beliefs in this context has been discussed by some authors (Czeisler et al., 2020; Ko et al., 2020) and HBM relevant variables such as risk perceptions have been shown to be on the minds of people across different cultural contexts (Iorfa et al., 2020; Sobków et al., 2020). We identified one contribution that relates health beliefs with health anxiety (Asmundson and Taylor, 2020). Overall, the potential of HBM has been clearly identified by a number of commentators, including for reinforcing behaviors that limit the spread of the virus (Carico et al., 2020), and for managing mental health concerns (Mukhtar, 2020). Focusing on empirical studies, a Polish study found that dark personality traits such as psychopathy correlated with health beliefs related to the COVID – 19 and undermined effective actions (Nowak et al., 2020). Another study Elgzar et al. (2020) found that HBM implemented within an educational program in Saudi Arabia increased students’ perceived susceptibility, severity, benefits and self-efficacy in overcoming perceived barriers in the adoption of protective and preventive behavior.

Clark et al. (2020) reported a study that directly aligns with our goals and assessed the contribution of various health beliefs on voluntary compliance with recommended preventive behaviors across seven countries, including Italy (Clark et al., 2020). They found that after controlling for demographics, the most important predictor of taking health precautions was self-efficacy, while perceived severity and susceptibility were of little importance. However, the authors did not assess how individuals perceived these beliefs and whether cultural dynamics may influence the performance of the HBM. Culture, perceptions and behavior are intrinsically linked, which makes cultural psychology indispensable when examining work with immediate real-world impact (Wang, 2016).

In summary, the HBM shows promise as a useful tool for COVID-19 relevant information and behavior change (Carico et al., 2020; Nowak et al., 2020), but little work has been done to examine effectiveness across different cultural contexts. We examine the HBM in a high stakes public health emergency, which alters the usual decision making environment in two different countries with different profiles at the time of measurement.

### The Case Studies Context

We focus on Italy and Romania because of their cultural characteristics and specific pandemic situation at the time of the data collection. The two countries are historically closely related, sharing a Romance language and long stretches of shared distal history. Yet, Romania was part of the former Soviet bloc, leading to divergent political and social conditions for more than 40 years. Consequently, the two countries currently have somewhat different cultural values with Italy being part of a Catholic European value cluster, whereas Romania is part of an Orthodox value cluster within Europe (World Values Survey, no date). The World Values Survey provides the most rigorous, representative and frequent analysis of cultural orientations on a global scale, with representative data going back to 1985 (Welzel and Inglehart, 2010). Two major dimensions have emerged that can be used to understand broad cultural dynamics (Inglehart and Baker, 2000). Italy and Romania differ primarily on the Survival vs. Self-Expression dimension, which differentiates an emphasis on security and a motivation to avoid threats vs. an orientation to life which takes survival for granted and prioritizes self-expression and quality of life. These value distinctions have been linked to basic needs that emerge within specific ecological and economic contexts (Van de Vliert, 2007; Welzel, 2013). This value polarity is also relevant for the control of disease threats, as it prioritizes free exploration vs. restrictions of personal impulses and is relevant for containing spread of infectious diseases (Schaller, 2011).

This cultural distinction becomes even more salient when seen within the context of demographic and social structures of the two countries. Romania has a public health care system that underperforms in many respects (Fărcăşanu, 2010; Ungureanu et al., 2017; Horodnic et al., 2018; Precupeţu and Popa, 2020). Therefore, individuals in Romania may feel more at risk given the lack of trust and acknowledged problems with the public health system. In contrast, Italy has a highly functional health care system. At the same time, Italy has a high share of elderly, with the percentage of people over 65 years being 22.1% (compared to 17.58% in Romania) (“Romania Demographics Profile, 2020). This likely has led to a greater casualty rates in Italy, as the elderly are the most vulnerable segment of the population (Hulíková Tesárková, 2020). Furthermore, Italy is characterized by extended families (Caserta et al., 2021), which facilitates contacts between young and old people, therefore accelerating likely transmission of the virus.

Italy was the first country in Europe, together with Germany, where the virus began to spread, starting from the end of January. In Italy the spread of the epidemic has been particularly rapid. Within 1 month, both the central government and regional governments started to adopt the first restrictive measures, isolating the areas of epidemic outbreak (the so-called red areas) and introducing increasing limits to people’s movements. At the beginning of March, the interruption of all economic activities and complete lockdown for all citizens were decreed by law. Despite this, the progression of the epidemic continued throughout the month of March, reaching 147,577 infected and 18,849 deceased by April 10, 2020 (Source: Italian Ministry of Health). In mid-March the number of new infected stopped growing and at the end of March, the number of deceased began to decline after reaching a peak of nearly 1,000 deaths per day.

At about a month after Italy confirmed its first cases, the virus reached Romania. However, over the first 2 weeks, the COVID-19 epidemic had a relatively slower evolution. The Romanian government started implementing several measures such as banning all public gatherings and international travels, closing schools, restaurants, cafes, shopping malls, limiting or prohibiting the movement of persons for no urgent reason and instituting a national lockdown to enforce these measures. In spite of these actions, the virus continued to spread throughout March and the beginning of April, reaching 5,990 confirmed cases of COVID-19, and 291 deceased. At the end of March, the number of deaths began to start growing, reaching the maximum of 28 deaths per day by the mid of April.

The different timing between the two countries in the development of the epidemic has led, in the case of Romania, to greater awareness on the severity of the effects of the contagion, following the news arriving from Italy. The greater cultural orientation toward survival values together with the lower average income and perceived weaker and less efficient health system (Popa et al., 2017; Druică et al., 2019; Cosma et al., 2020) may have led to a greater level of attention in the Romanian population, and therefore the adoption of more careful prevention behaviors. Conversely, the Italian population seems to have initially underestimated the risks associated with COVID-19, adopting less rigorous preventive behaviors based on values of self-expression and relying on a health care system that was perceived to be among the most qualified within international comparisons (Björnberg and Phang, 2019; Motta Zanin et al., 2020).

### The Study Goal

Our study had three major goals: (1) to examine the applicability and effectiveness of the health beliefs model to understand individual’s prevention behavior during an acute public health crisis, (2) using a cultural psychology lens we explicitly test the HBM in two cultural context that vary both in level of threat and the salience of survival values and (3) to examine individual differences within these two contexts, that is identify what demographic groups are particularly diligent in following these behaviors. Overall, our study provides important new insight on the effectiveness of HBM variables for improving health behaviors, which can help with improving communication targets and pathways about COVID-19 in the ongoing pandemic.

## Materials and Methods

### Sampling Methodology

We collected our data via a combination of open email-based and web-based survey, distributed between March 13 to March 27, 2020 in Romania and from March 18 to April 1st, 2020 in Italy. Invitations were disseminated through Facebook, LinkedIn, WhatsApp, and other social networks, as well as via email networks. The Center of Applied Behavioral Economics, University of Bucharest, and Carlo Bo University of Urbino, Italy jointly conducted the study. The respondents were informed at the beginning of the survey that their participation is voluntary and anonymous and that by completing the questionnaire, they provide consent to participation in this study.

The sampling methodology was based on chain-referral sampling (Biernacki and Waldorf, 1981), by adopting a non-probabilistic snowball process, which is based on contacting one participant via the other (Browne, 2005). This method allows to quickly improve the scope of on-line questionnaires and optimizes the balance between time and costs (Baltar and Brunet, 2012). Differently from the respondent-driven sampling (RDS) (Heckathorn, 2011a,b), the respondents have not been traced in the recruitment waves following the initial seeds of respondents, and they did not receive any material compensation or prize for their participation in the research.

The initial seeds of the samples have been chosen by convenience and not randomly, with self-selected participants opting in based on their availability to answer the questionnaire. Participants were asked to pass the questionnaire to their social networks, thus identifying new groups of respondents and exponentially growing the size of the sample. Although convenience sampling is often criticized for not providing representative samples and thus running the risk of biased results due to the non-representative nature of the Internet population and any volunteer effects (Eysenbach and Wyatt, 2002; Schonlau, 2004), it is important to define for which subset of a population the conclusions drawn from a convenience sample are assumed to be valid (Eysenbach, 2004) and hence, the interpretation and conclusions need to be discussed with these constraints in mind.

### Participants

A total of 1,868 respondents (1,126 individuals from Romania and 742 individuals from Italy) provided valid answers. The average age was 33.89 (*SD*: 13.25, Range: 16–82) in Romania, which was significantly higher compared to the average age in the Italian sample: 36.94 (*SD*: 15.07, Range: 14–79), *t*(1442.7) = 4.487, *p* < 0.001. This age difference is aligned with the overall age distribution of the two countries (Romania Demographics Profile, 2020). Further, significantly more participants in the Italian sample were male (38.14%) compared to the Romania sample (24.51%). A comparable number of individuals were married, with the overall rate being 70% (70.78% in Romania, 68.87% in Italy). The number of individuals with children was somewhat higher in Romania (38.54%) compared to Italy (35.58%). A significantly higher number of respondents were medical students in Romania (14.12%) compared to Italy (7.14%). Although the sample is not fully representative of the characteristics of the population due to the sampling method adopted, the overall sample composition approximates the general population. We include the demographic variables in our models described below, which allows us to statistically control for any demographic differences. Detailed demographics and statistical comparisons between the samples can be found in Table 1.

**TABLE 1:** Descriptive statistics for Romania and Italy.

	Romania	Italy	Difference
Male	276(24.51%)	283(38.14%)	χ^2^(1) = 38.969, *p* < 0.001
Student	416(36.94%)	277(37.33%)	χ^2^(1) = 0.014, *p* = 0.904
Medical background	159(14.12%)	53(7.14%)	χ^2^(1) = 20.958, *p* < 0.001
Married or partnership	797(70.78%)	511(68.87%)	χ^2^(1) = 0.692, *p* = 0.406
Parents	434(38.54%)	264(35.58%)	χ^2^(1) = 1.555, *p* = 0.212
Higher education	763(67.76%)	483(65.09%)	χ^2^(1) = 1.316, *p* = 0.251

### Measurement

#### Health Beliefs

Health beliefs were measured with a 24-item Likert-scale ranging from 1 (totally disagree) to 7 (totally agree). The health beliefs scale was previously used to measure the following belief dimensions (Hartley et al., 2018): Perceived susceptibility to the illness (four items, one item was excluded in our study due to differential translations in Romania and Italian), perceived severity of the illness (eight items), perceived benefits of preparing against the illness (three items), perceived barriers to preparation (five items), perceived self-efficacy (four items). The complete list of items is available in the Appendix, while the reliability of the individual measures in Romania and Italy are presented in Table 2.

**TABLE 2:** Reliability of the individual measures in Romania and Italy along with the corresponding 95% confidence intervals.

Measure	α	ω
	Romania| Italy	Romania| Italy
Susceptibility	0.780[0.759, 0.801]| 0.814[0.792, 0.836]	0.794[0.775, 0.813]| 0.830[0.811, 0.850]
Severity	0.878[0.867, 0.889]| 0.879[0.866, 0.892]	0.869[0.857, 0.882]| 0.860[0.845, 0.876]
Benefits	0.524[0.482, 0.566]| 0.862[0.845, 0.879]	0.537[0.493, 0.581]| 0.862[0.845, 0.880]
Barriers	0.751[0.729, 0.772]| 0.634[0.600, 0.669]	0.750[0.726, 0.773]| 0.638[0.598, 0.679]
Self-efficacy	0.870[0.858, 0.883]| 0.790[0.766, 0.814]	0.871[0.858, 0.883]| 0.793[0.768, 0.817]

*Values are listed as Romania| Italy.*

#### Health Behavior

To assess participants behavior we asked them about their adoption of 8 commonly recommended prevention behaviors at the time of our study (Washing hands, cleaning surfaces with alcohol regularly, etc.). Participants answered on a 1–7 scale. The reliability of all measures [including ω, GLB as alternatives to αααα (Trizano-Hermosilla and Alvarado, 2016)] can be found in Table 2 and correlations between the health belief facets in Table 3.

**TABLE 3:** Correlation of the health belief facets in Romania and Italy.

Measure	N	M	*SD*	1	2	3	4
	Romania| Italy	Romania| Italy	Romania| Italy	Romania| Italy	Romania| Italy	Romania| Italy	Romania| Italy
Self-efficacy	1126| 742	6.04| 4.74	1.03| 1.37				
Susceptibility	1126| 742	3.41| 3.19	1.3| 1.28	0.06*| 0.19**			
Benefits	1126| 742	5.41| 5.51	1.18| 1.45	0.41**| 0.50**	0.08*| 0.22**		
Barriers	1126| 742	1.95| 1.92	1.07| 0.91	−0.33**| −0.01	0.14**| 0.18**	−0.09**| −0.03	
Severity	1126| 742	3.29| 3.47	1.5| 1.4	0.02| 0.23**	0.34**| 0.40**	0.17**| 0.37**	0.27**| 0.15**

Values are listed as Romania| Italy; *p = 0.05, **p = 0.01, ***p = 0001.

#### Demographics

We included the following demographics: age, gender (0 = female, 1 = male), student (0 = no, 1 = yes), medical studies undertaken (0 = no, 1 = yes), in a relationship (0 = no, 1 = yes), parent (0 = no, 1 = yes), higher degree (0 = No, 1 = Yes), and chronic patient (0 = no, 1 = yes).

### Statistical Analysis

First, we assessed the equivalence of the health beliefs scale across Romania and Italy, by using confirmatory factor analysis in an attempt to identify a unique, and invariant model in both samples. Considering that the Romanian sample was larger than the Italian sample, first, we identified the best-fitted model in Romania that was then fitted across both samples. We assessed whether the model shows a similar structure across samples, tested for metric equivalence (similarity of loadings) and scalar invariance (similarity of intercepts) (Fischer and Karl, 2019).

Second, to test whether endorsement differed across behaviors and countries we fitted a General Linear Model in which endorsement was predicted by behavior, country, their interaction (to test for differential effectiveness across the two sample locations), and a random effect for participants.

Third, we tested the effect of demographics and health beliefs on prevention behaviors by using a multi-group path model, in which each behavior was predicted by the observed health belief variables and the demographics. We subsequently constrained all regression paths to be equal for Romania and Italy to increase the parsimony of the model and allow for easier interpretation (Fischer and Karl, 2019). A separate model in which we used the full latent model is reported in the Supplementary Material on the OSF. Overall, the results were comparable, with the major differences being that the path between latent perceived benefits and disinfecting surfaces did no longer significantly differ between countries, but the path between latent perceived barriers and washing hands did vary between countries.

## Results

### Model Equivalence Across Countries

The model in Romania showed good fit (CFI = 0.916, RMSEA = 0.060 [0.057, 0.064], SRMR = 0.066) after we introduced a covariance between the three severity items “When I think of Coronavirus, my heart starts beating faster” and “I am afraid to think about Coronavirus,” “The thought of getting sick with Coronavirus scares me” (indicating the possible presence of an anxiety factor in the severity measure) and between the self-efficacy items “I know how to adopt a preventative behavior when it comes to getting sick with Coronavirus” and “I am confident that I can properly adopt a preventive behavior regarding Coronavirus disease.”

**FIGURE 1:**
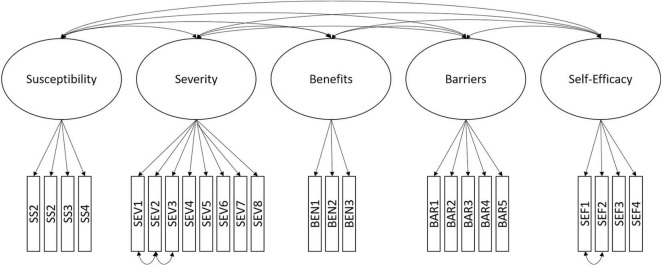
Conceptual model of the final CFA structure.

We subsequently fitted this model across both samples and found good configural fit, as well as metric invariance but not scalar invariance (see Table 4). This is a first important outcome from a cultural perspective; individuals in the two samples perceived and interpreted the constructs in a similar manner. Overall, this indicates that the current measurement model of the HBM works sufficiently well to explore the relationship with other variables across countries, but we are not in a position to compare mean differences with this measure, but only relative endorsement of perceptions (e.g., profiles). A conceptual representation of the model is shown in Figure 1 and all item loadings constrained across countries can be found in Supplementary Table 1.

**TABLE 4:** Model fit across levels of equivalence.

CFI	RMSEA	SRMR	Δ CFI	Δ RMSEA	Interpretation
0.912	0.063[0.060]	0.066			Configural equivalence, structure comparable
0.907	0.063[0.061, 0.066]	0.069	0.005	−0.001	Metric equivalence, loadings and relationships/correlations comparable
0.811	0.089[0.086, 0.091]	0.095	0.096	−0.025	No scalar equivalence, means not comparable

### Prevalence of Health Behaviors Across the Two Samples

**FIGURE 2:**
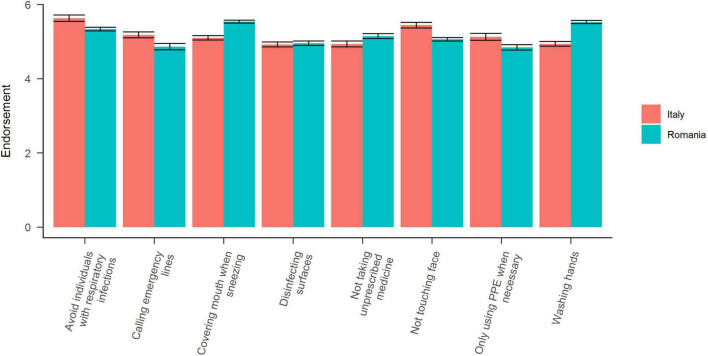
Self-reported practice of behaviors aimed at reducing the spread of COVID-19. All error bars represent 95% CI corrected for within-subjects comparisons. All behaviors were standardized within participants and normalized across countries to increase the interpretability.

Overall, we found significant differences based on country [*F*(1,14928) = 10, 538.26, *MSE* = 3.27, *p* < 0.001], behavior [*F*(7,14928) = 22.57, *MSE* = 3.27, *p* < 0.001], and their interaction [*F*(7,14928) = 19.43, *MSE* = 3.27, *p* < 0.001]. In Romania the three most endorsed behaviors were: Avoiding contact with individuals that show respiratory symptoms, not touching one’s face, and calling emergency lines when experiencing fevers or coughs. The least endorsed behaviors in Romania were: Disinfecting surfaces, not taking non-prescribed medicine, and washing hands. In Italy the three most endorsed behaviors were: Covering one’s mouth/nose while sneezing our coughing, washing hands, and avoiding contact with individuals that show respiratory symptoms. The least endorsed behaviors in Italy were: Only using PPE when necessary, Calling emergency lines, and disinfecting surfaces (we show the results for both countries in Figure 2).

### The Effect of Demographics on Prevention Behaviors

**FIGURE 3:**
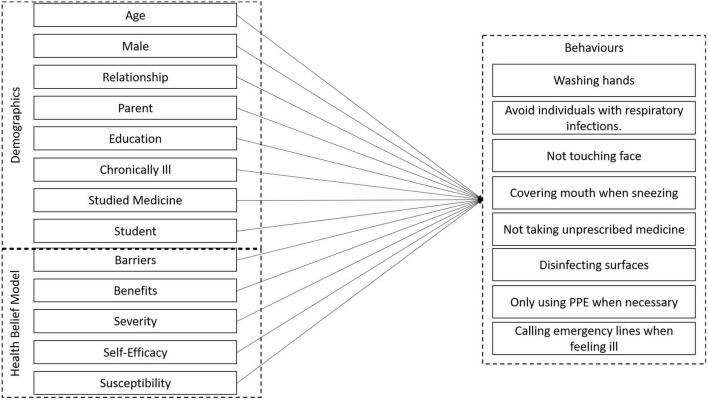
Conceptual representation of the path-model with all behaviors entered simultaneously.

We fitted a model in which the health beliefs predicted the individual behaviors, with the paths constrained across countries with a MLM estimator. The model showed excellent fit to the data (CFI = 0.99, RMSEA = 0.031 [0.024, 0.037], SRMR = 0.03). To investigate country differences, we examined the expected χ^2^ change for each path if it would be released and estimated separately across countries. We selected the path with the highest expected χ^2^ change in the fully constrained model and subsequently adjusted all other *p*-values using a Bonferroni correction based on the number of previously selected paths. Overall, we released 7 paths. The following paths were released in this order:

(1) Path between covering mouth when sneezing and self-efficacy (χ^2^ = 13.994, *p*_adj_ < 0.001),

(2) Covering mouth when sneezing and perceived benefits (χ^2^ = 13.335, *p*_adj_ < 0.001),

(3) Disinfect surfaces and perceived benefits (χ^2^ = 13.222, *p*_adj_ < 0.001),

(4) Disinfect surfaces and self-efficacy (χ^2^ = 9.207, *p*_adj_ = 0.008),

(5) PPE usage and perceived benefits (χ^2^ = 8.965, *p*_adj_ = 0.015),

(6) Washing hands and age (χ^2^ = 8.389, *p*_adj_ = 0.024), and

(7) Washing hands and parental status (χ^2^ = 7.989, *p*_adj_ = 0.035). We report all constrained and unconstrained paths in Table 5 and show a conceptual representation of the model in Figure 3.

**TABLE 5:** Model Results for the SEM path-model across countries.

	Washing hands	Avoid individuals with respiratory infections	Not touching face	Covering mouth when sneezing	Not taking unprescribed medicine	Disinfecting surfaces	Only using PPE when necessary	Calling emergency lines when feeling ill
Age	0.*002* *[−0.005, 0.009]*| *0.007* *[−0.001, 0.015]*	0 [−0.007, 0.006]	0.003 [−0.004, 0.01]	0 [−0.006, 0.006]	0.005 [−0.002, 0.012]	0.005 [−0.003, 0.012]	0.005 [−0.005, 0.015]	0 [−0.009, 0.009]
Male	−0.128 [−0.221, −0.035]**	−0.049 [−0.165, 0.068]	−0.213 [−0.342, −0.085]**	−0.106 [−0.196, −0.015]*	−0.25 [−0.384, −0.116]***	−0.214 [−0.344, −0.084]**	−0.128 [−0.287, 0.031]	−0.159 [−0.321, 0.003]
Relationship	0.069 [−0.016, 0.153]	0.024 [−0.088, 0.137]	0.057 [−0.068, 0.182]	−0.001 [−0.085, 0.082]	0.159 [0.024, 0.294]*	0.154 [0.021, 0.287]*	0.044 [−0.127, 0.214]	0.13 [−0.045, 0.304]
Parent	*−0.044* *[−0.154, 0.066]*| *0.068* *[−0.108, 0.244]*	−0.014 [−0.139, 0.111]	−0.079 [−0.224, 0.066]	0.052 [−0.049, 0.153]	0.017 [−0.136, 0.169]	−0.076 [−0.227, 0.075]	−0.043 [−0.259, 0.172]	0.026 [−0.177, 0.23]
Education	0.059 [−0.045, 0.162]	−0.06 [−0.187, 0.066]	0.042 [−0.088, 0.173]	0.02 [−0.078, 0.118]	0.015 [−0.133, 0.163]	−0.084 [−0.223, 0.055]	−0.07 [−0.245, 0.105]	−0.098 [−0.269, 0.073]
Chronically Ill	−0.011 [−0.134, 0.113]	−0.056 [−0.192, 0.081]	−0.096 [−0.252, 0.059]	−0.09 [−0.212, 0.032]	−0.028 [−0.191, 0.136]	−0.116 [−0.275, 0.043]	−0.194 [−0.412, 0.024]	−0.061 [−0.288, 0.166]
Studied medicine	−0.078 [−0.179, 0.024]	−0.306 [−0.466, −0.146]***	0.025 [−0.115, 0.166]	−0.023 [−0.101, 0.056]	−0.036 [−0.194, 0.123]	0.078 [−0.081, 0.237]	−0.218 [−0.452, 0.015]	−0.153 [−0.374, 0.069]
Current student	0.087 [−0.027, 0.201]	−0.029 [−0.173, 0.114]	0.117 [−0.035, 0.269]	−0.005 [−0.118, 0.109]	−0.03 [−0.204, 0.144]	−0.075 [−0.238, 0.087]	0.004 [−0.203, 0.212]	0.045 [−0.164, 0.254]
Barriers	−0.059 [−0.114, −0.005]*	−0.058 [−0.116, −0.001]*	−0.07 [−0.136, −0.004]*	−0.033 [−0.078, 0.013]	−0.067 [−0.134, 0.001]	−0.041 [−0.102, 0.02]	−0.054 [−0.134, 0.026]	−0.085 [−0.161, −0.009]*
Benefits	0.058 [0.017, 0.1]**	0.077 [0.03, 0.123]**	0.093 [0.038, 0.147]***	0.*045* [0.012, 0.079]**| *0.095* [0.029, 0.161]**	0.064 [0.004, 0.123]*	0.*143* [0.07, 0.217]***| *0.038* *[−0.03, 0.105]*	*0.218* [0.131, 0.304]***| *0.066* *[−0.013, 0.145]*	0.099 [0.032, 0.165]**
Severity	0.038 [0.016, 0.06]***	0.064 [0.03, 0.099]***	0.065 [0.026, 0.104]**	0.018 [−0.003, 0.039]	−0.011 [−0.052, 0.031]	0.097 [0.052, 0.142]***	0.025 [−0.029, 0.08]	0.086 [0.026, 0.146]**
Self*-*efficacy	0.19 [0.121, 0.259]***	0.212 [0.142, 0.282]***	0.222 [0.148, 0.296]***	0.*126* [0.061, 0.191]***| *0.197* [0.116, 0.278]***	0.194 [0.119, 0.27]***	*0.228* [0.14, 0.317]***| *0.167* [0.083, 0.251]***	0.192 [0.108, 0.277]***	0.149 [0.065, 0.233]***
Susceptibility	−0.011 [−0.042, 0.019]	0.009 [−0.033, 0.051]	−0.003 [−0.049, 0.043]	−0.013 [−0.043, 0.018]	0.026 [−0.025, 0.076]	−0.016 [−0.062, 0.03]	−0.02 [−0.079, 0.04]	−0.014 [−0.079, 0.05]

All values are unstandardized B with 95% confidence intervals. Values that are unconstrained across countries are reported as Romania| Italy and are reported in italics; *p = 0.05, **p = 0.01, ***p = 0.001.

Focusing on the demographic effects that were similar across countries, only gender, medical studies background, and relationship status showed significant effects. Male participants (compared to female participants) were less likely to wash their hands *B* = −0.128[−0.221, −0.035], *p* = 0.007, not touch their faces *B* = −0.213[−0.342, −0.085], *p* < 0.001, to cover their mouth when sneezing *B* = −0.106[−0.196, −0.015], *p* = 0.022, not take non-prescribed medicine *B* = −0.25[−0.384, −0.116], *p* < 0.001, and disinfect surfaces *B* = −0.214[−0.344, −0.084], *p* = 0.001. In contrast, participants in a relationship (compared to single participants) were more likely to not take unprescribed medicine *B* = 0.159[0.024, 0.294], *p* = 0.021 and disinfect surfaces 154[0.021, 0.287], *p* = 0.023. Finally, participants with medical studies background were more likely to avoid individuals with respiratory illnesses *B* = −0.306[−0.466, −0.146], *p* < 0.001.

### The Effect of Health Beliefs on Prevention Behaviors

Regarding the individual components of HBM we found that perceived self-efficacy was a significant predictor of all behaviors. It was the only part of the model that consistently emerged as a significant predictor for each recommendation. It was also the strongest predictor in absolute terms (examining the size of the unstandardized path coefficients). Concerning differences between samples, self-efficacy was a significantly stronger predictor for covering one’s mouth when sneezing in Italy compared to Romania, but disinfecting surfaces was more strongly associated with self-efficacy in Romania compared to Italy. Perceived benefits also significantly predicted all behaviors in Romania (and all but two of the behaviors in Italy), but the relative strength of the relationship was weaker compared with perceived self-efficacy. Concerning the differences between the two samples, perceived benefits were again more strongly related to covering one’s mouth when sneezing in Italy compared to Romania; whereas benefits were not significant to disinfecting surfaces in Italy and was significantly and substantively correlated with perceived benefits in Romania. Finally, the use of protective equipment only when needed was associated with benefits in Romania, but not in Italy. Perceived barriers and severity significantly correlated with four of the behaviors with about equal strength: washing hands, avoiding individuals with respiratory infections, not touching one’s face and calling emergency lines when feeling ill. In addition, severity was positively associated with disinfecting surfaces, but perceived barriers were not. The only belief in the HBM that did not correlate with any behaviors after controlling for the other beliefs was susceptibility.

### Exploration of Mediation Models

As highlighted by a reviewer, the revised HBM includes mediation effects of demographic variables on health behaviors via the main HBM variables (Glanz et al., 2008; Jones et al., 2015). In other words, demographic effects such as age or gender should only influence health behavior via central variables within the HBM. We explored these options in our data and provide full results in the Supplementary Material. We set up independent models in each sample. The demographic variables of age, gender and medical background were included as exogeneous variables. The core variables of the HBM (perceived susceptibility to the illness, perceived severity of the illness, perceived benefits, perceived barriers, and perceived self-efficacy) were included as potential mediators. The behavioral items were included as outcomes. A full description of our analytical procedure is also included in the Supplementary Material. The main results from this exploration suggested that: (a) gender effects on washing hands, avoiding individuals with respiratory infections, not touching one’s face and disinfecting surfaces were mediated by perceived severity (with males reporting lower intentions to perform the behavior mediated via reduced severity) and these effects were not statistically different across the two samples; (b) age effects on all behaviors were mediated by perceived benefits in Romania, but (c) not in Italy. Older Romanians were more likely to perform these behaviors and this was mediated via greater perceived benefits. There were also weaker indirect effects of age on all behaviors via self-efficacy, with older individuals more likely to perform behaviors via greater self-efficacy. Finally, individuals with a medical background were more likely to perform these protective behaviors. The relation was mediated via greater self-efficacy, irrespective of sample background. Medical background was also positively related to washing hands, not touching ones face, covering the mouth when sneezing, not taking unprescribed medicine and calling emergency lines via perceived benefits, again irrespective of sample. Therefore, perceived benefits and self-efficacy appear to be better mediators of age and medical background demographics, while perceived severity mediated the effects of gender on preventive behaviors. Full information is provided in the Supplementary Material.

## Discussion

The goal of our study was to use tools from cultural psychology to examine the Health Belief Model during the COVID – 19 pandemic in two samples that are characterized by different levels of infection and differential emphasis of survival vs. self-expression values. First of all, we found that the core variables of the HBM were perceived similarly in the two cultural contexts, but there were baseline differences that preclude direct comparisons between the two samples. This is a first crucial step in any cultural analysis as the outcomes of this analysis determine how results can be interpreted (Fontaine, 2011). In our case, we can safely compare the effectiveness of the model across the two contexts, but we cannot directly compare the base rates.

We found that there was no single behavior that was widely adopted in both samples. At the time of our study, there was still no strong consensus in the literature on specific protective behaviors, beyond increased personal hygiene and covering one’s face when sneezing. Not surprisingly, given the diversity of medical opinion, our participants reported a number of diverse behaviors and there was no clear and consistent pattern across both samples. Romanian people received daily updated news from the media on the progress of the epidemic in China and then in Italy. Given the cultural preoccupation with security, this seems to have stimulated greater adoption of preventive behaviors prior to the start of the epidemic in their country. In turn, the adoption of preventive behaviors may have contributed to slowing the spread of the epidemic, avoiding the rapid increases experienced in Italy. Although we cannot directly compare the individual behavior items, the overall means were much higher in Romania compared to Italy. This may be driven by the combination of a cultural orientation emphasizing security with the news of the negative impact of the pandemic in nearby Italy.

The exploration of individual differences is important within a cultural psychology perspective (Wang, 2016). We found that women overall were more likely to adopt protective behaviors. These patterns are in line with the overall pattern reported in the literature, suggesting that men are more likely to take risks and less likely to seek medical help compared to women (Byrnes et al., 1999; Nam et al., 2010). Age influenced health behaviors via perceived benefits and self-efficacy in Romania, but not in Italy. Older individuals are typically more strongly acculturated (Taras et al., 2010), suggesting that cultural dynamics on behaviors via salient health perceptions may more strongly operate in Romania vs. Italy. This is in line with recent evidence of differential norm strength in the context of the pandemic (Fischer and Karl, 2021; Gelfand et al., 2021).

### Theoretical Implications

We explicitly tested the properties of current HBM instruments across two cultures. Any cultural exploration depends on the validity of the data (Fontaine, 2011; Smith et al., 2013; Wang, 2016). Our model overall fitted well across both samples and the association between individual items and the overall constructs was comparable. From a cultural perspective, this implies that individuals have comparable conceptualizations of salient health beliefs in these two contexts.

When examining the specific patterns, we found that perceived self-efficacy – that is the belief of being able to successful protect oneself from being infected – was the most consistent and strongest statistical predictor of health behaviors. This supports general findings in the wider psychological literature that self-efficacy is crucial for understanding behavior and behavior change (Rosenstock et al., 1988; Wang and Zhang, 2016). The second most consistently associate health belief was perceived benefit. This fits with the larger literature (Bond et al., 1992) and implies that individuals are more likely to adopt preventive behaviors that are seen as beneficial for individuals. Perceived barriers and severity also showed some effects in both samples, but overall were less strongly associated. In contrast, perceived threat may not be sufficient to motivate behavior in the absence of a belief to be able to protect oneself through adopting effective measures. These results align with the findings of Janz and Becker (1984) who researched the effectiveness of health beliefs on the adoption of preventive behaviors in a wide variety of contexts. However, the absence of a threat effect needs to be more thoroughly investigated, including in longitudinal studies.

Concerning cultural differences in the strengths of associations, we found relatively few differences compared to the largely consistent patterns for the HBM variables across the various behaviors. On one hand, the two settings share many cultural features, with the major difference being along the survival vs. self-expression value dimension. For the Italian sample, it seems that salient behaviors (covering one’s mouth) were better predicted by perceived efficacy and benefits; whereas the least endorsed behavioral actions were less well predicted by these HBM variables. These findings align with previous literature showing that how HBM factors relate in terms of weights and predictive power may vary with target behaviors (Abraham and Sheeran, 2005), and that some HBM factors can be more effective than others in explaining adherence to specific behaviors in concrete interventions (LaBrosse and Albrecht, 2013; Jones et al., 2014). Our pattern suggests that health belief variables are better predictors of individually focused, but more frequent behaviors in the Italian context. This may align with the self-expression values that are comparatively more salient in Italy – individuals perform those behaviors that can be easily performed and are seen beneficial and easy to perform for the individual. In contrast, in our Romanian sample disinfecting surfaces were among the least endorsed behaviors but were also somewhat better predicted by health beliefs compared to our Italian sample. Given the greater concern with security in Romanian society, the beliefs of the effectiveness of this behavior may have led to this stronger behavioral association.

These patterns suggest that normatively shared beliefs within a population are important for understanding the adoption of health behaviors, which have follow-on effects for the larger social and cultural system (Daniel et al., 2021; Fischer et al., 2021). As we have seen in the first stages of the pandemic in Northern Italy, the impact of the pandemic on social and cultural conditions due to extended lockdowns may be substantive.

Looking more broadly at the emerging patterns in different contexts, our findings concur with emerging findings using the HBM in other countries. The HBM dimensions were correlated with preventive behavior in India, however, the infection risk as perceived by the respondents was not the same as actual risk (Jose et al., 2021). Focusing on individual differences, research in Brazil showed gender, income and health status effects on the link between both perceived susceptibility and severity on preventive behavior (Costa, 2020, p. 202). An Iranian study on adult population found that after controlling for gender and residence, the strongest predictors of preventive behavior against COVID-19 were perceived barriers, perceived self-efficacy, fatalistic beliefs, and perceived interests (Shahnazi et al., 2020), whereas a second Iranian study conducted with adolescents found that the strongest predictor of COVID preventive behavior was self-efficacy (Fathian-Dastgerdi et al., 2021). A Chinese study found that HBM variables were correlated with preventive behavior but that the magnitude of correlations were small (Tong et al., 2020). In Ethiopia, self-efficacy, perceived benefits, perceived barriers, and perceived susceptibility of COVID-19 as well as cues to action correlated with preventive behaviors (Tadesse et al., 2020; Yehualashet et al., 2021). Together with these other studies, our research suggests that HBM is a useful framework, but the variability also implies that cultural dynamics play a role and need greater attention. Possible candidates for further exploration include social axioms (e.g., Tong et al., 2020), personality dynamics (Nowak et al., 2020; Fischer et al., 2021), and the role of emotions in the cultural shaping of COVID-19 narratives (Chentsova-Dutton, 2020).

### Practical and Managerial Implications

A fact that clearly emerges from the study is that the greater awareness of the severity of COVID-19 correlates with more prudent behavior by the population. This has significant implications for information policies regarding the development of a pandemic with serious consequences such as COVID-19. In the case of Italy, some mistakes were made, since communication policies to the population were initially contradictory: on the one hand, people were invited to follow preventive behavior, on the other, they were encouraged not to abandon normal habits due to the risk of a slowdown in several economic sectors (especially travels, restaurants, and retailing) (De Blasio and Selva, 2021). For example, on February 27, the mayor of Milan launched an advertising campaign on social networks entitled “Milan doesn’t stop,” with famous people depicted while drinking in a bar. This means that in the face of a pandemic of proven serious threat, communication by the authorities must be clear and unambiguous, giving priority to the safety of people before safeguarding economic interests. To instill optimism in such situations can be deleterious, and communication should emphasize the risks rather than understate them. Our results suggest that we need different emphasis in the contents of the communication (as relevant within HBM). In particular, the content of health communications may aim to emphasize perceived efficacy especially in contexts where efficacy beliefs are weaker, but communicators may also consider the perceived degree of threat posed by the disease. In addition, the source of health communication should be appropriate to the cultural context (for an example highlighting the role of religious leaders see: Osei-Tutu et al., 2021a). In Italy, the initial high confidence in the national healthcare system may have led to underestimation of the risks of the pandemic, and this suggests that in the face of diseases with unknown seriousness and harmfulness, it is important to adopt a prudent attitude by emphasizing the potential dangers rather than downplaying them.

## Limitations

One clear limitation of our current study is the convenience nature of our sample. A further limitation is the self-reported nature of the behaviors, which might be susceptible to response bias and reference group effects (Heine et al., 2002). The means on all measures were consistently higher in Romania compared to Italy. This pattern may suggest some ceiling effects in the former country compared to the latter and possible reference group effects (Heine et al., 2002). The disease context may influence both behavioral compliance rates and the perceptions of compliance rates which influences self-reports of the behavior. Absent more objective indicators, we cannot disentangle response set and substantive processes. A third limitation is that the countries followed different communication strategies about preventive behavior. This is of theoretical importance because it may trigger action cues which has been discussed as a moderator of HBM. We focus on the direct effects of the HBM in our study, yet these effects might be modulated by specific cues to action, which could be explored in future research. A fourth limitation from a cultural comparative perspective is that we did not include specific measures of cultural values. Unfortunately, the rapidly developing situation during the early stages of the pandemic together with pragmatic constraints on the number of instruments that could be included in an online study, we were unable to include measures of cultural values. Future studies on the HBM including measures of cultural values and norms are highly encouraged. Related to this point, our approach was focused on beliefs by individuals in two specific contexts, which does not allow a differentiation of individual vs. group-level normative processes within the context of these behaviors. Future research clearly needs to start examining the intersection between individual and group-level processes (for one possible example using sample level processes, see Fischer and Karl, 2021). Finally, in our current study we focus on cognitive factors as part of the HBM, it is nevertheless likely that emotional and affective responses to COVID-19 shape individuals prevention behavior which could be examined as potential moderators or mediators in future studies (Daniel et al., 2021; Fischer et al., 2021).

Despite these limitations, our paper provides a snapshot of the endorsement of health behaviors in the acute context of the COVID – 19 crisis. It is important to gain insights into health behavior at the moment when those behaviors are crucial for containing further spread of the virus. The results imply that self-efficacy is an important contributor but also point to the importance of the perceived severity of the infection at the time of measurement. With only two samples measured at a single time point, it is not possible to disentangle time and context effects, especially considering that Italy and Romania varied in both central cultural values and severity of the pandemic. Future studies with more measurement points over time or a larger number of study sites that vary systematically in cultural orientations and include measures of cultural values and norms would be informative for examining the impact of disease context on the adoption of health behaviors.

## Conclusion

Overall, our study shows that the Health Belief Model can be used to understand what beliefs are associated with reporting appropriate health behaviors. At a practical level, this opens up important avenues for potential intervention programs for increasing adaptive health behaviors in early stages of a pandemic. The results show the importance of increasing self-efficacy and perceived benefits in order to convince people to take actions to limit the spread of a new virus. From a cultural psychology perspective, the relative divergence for some of the variables also points to the need to study how individual health belief facets vary across countries and behaviors. We found that core constructs within the HBM were perceived similarly across these two contexts, but that means could not be directly compared. This highlights the importance of examining HBM more carefully across different cultural, social and economic contexts and the need to tailor interventions and communication about preventive measures to the specific context.

## Data Availability Statement

The datasets presented in this study can be found in online repositories. The names of the repository/repositories and accession number(s) can be found below: https://osf.io/k93dr/?view_only=fb66c152ffba45219451b4d03b4ba1e8.

## Ethics Statement

The studies involving human participants were reviewed and approved by the Ethics Committee of the University of Bucharest. The patients/participants provided their written informed consent to participate in this study.

## Author Contributions

ED, AS, and FM: data collection. ED, JK, RF, and AS: conceptualization. JK, RF, and ED: writing the original draft. ED, RF, FM, and JK: writing revised drafts. All authors contributed to the article and approved the submitted version.

## Conflict of Interest

The authors declare that the research was conducted in the absence of any commercial or financial relationships that could be construed as a potential conflict of interest.

## Publisher’s Note

All claims expressed in this article are solely those of the authors and do not necessarily represent those of their affiliated organizations, or those of the publisher, the editors and the reviewers. Any product that may be evaluated in this article, or claim that may be made by its manufacturer, is not guaranteed or endorsed by the publisher.
