# Supplementary material for: Testing the Effectiveness of the Health Belief Model in Predicting Preventive Behavior During the COVID-19 Pandemic: The Case of Romania and Italy
Source: Front Psychol. 2022 Jan 12;12:627575. doi: 10.3389/fpsyg.2021.627575 (PMC8789680; doi:10.3389/fpsyg.2021.627575)
Supplement: Supplementary file 1 [file Table_1.docx]

**Supplementary Material**

| Supplementary Table 1  Item loadings in the final CFA model with all loadings constrained to be equal across countries. | | | | |
| --- | --- | --- | --- | --- |
| Latent Factor | Item | Unstandardized Loading | Lower CI | Upper CI |
| Susceptibility | SS2 | 1.090 | 1.015 | 1.164 |
| Susceptibility | SS3 | 1.055 | .982 | 1.128 |
| Susceptibility | SS4 | 1.517 | 1.439 | 1.595 |
| Susceptibility | SS5 | .975 | .891 | 1.060 |
| Severity | SEV1 | 1.138 | 1.051 | 1.226 |
| Severity | SEV2 | 1.040 | .946 | 1.135 |
| Severity | SEV3 | 1.088 | .997 | 1.179 |
| Severity | SEV4 | 1.326 | 1.246 | 1.406 |
| Severity | SEV5 | 1.600 | 1.512 | 1.687 |
| Severity | SEV6 | 1.508 | 1.422 | 1.594 |
| Severity | SEV7 | 1.703 | 1.626 | 1.780 |
| Severity | SEV8 | 1.401 | 1.315 | 1.486 |
| Barriers | BAR1 | .870 | .752 | .988 |
| Barriers | BAR2 | .897 | .777 | 1.016 |
| Barriers | BAR3 | 1.046 | .933 | 1.159 |
| Barriers | BAR4 | 1.062 | .952 | 1.172 |
| Barriers | BAR5 | .819 | .709 | .930 |
| Benefits | BEN1 | .938 | .850 | 1.026 |
| Benefits | BEN2 | .921 | .833 | 1.008 |
| Benefits | BEN3 | .878 | .795 | .960 |
| Self-Efficacy | SEFF1 | .823 | .746 | .900 |
| Self-Efficacy | SEFF2 | .924 | .848 | 1.000 |
| Self-Efficacy | SEFF3 | 1.026 | .945 | 1.106 |
| Self-Efficacy | SEFF4 | .956 | .876 | 1.036 |
