# Supplementary material for: Testing the Effectiveness of the Health Belief Model in Predicting Preventive Behavior During the COVID-19 Pandemic: The Case of Romania and Italy
Source: Front Psychol. 2022 Jan 12;12:627575. doi: 10.3389/fpsyg.2021.627575 (PMC8789680; doi:10.3389/fpsyg.2021.627575)
Supplement: Supplementary file 2 [file Table_2.docx]

Exploratory Mediation Analyses

Following suggestions by one anonymous reviewer, we conducted exploratory mediation analyses. In a first step, we conducted a mediation path analysis, with separate analyses for each outcome behavior. We included age, gender (coded as male = 1) and medical education (coded as yes = 1) in the analyses. The individual results (see ST2) indicated that self-efficacy and benefits were the most important mediators for age and medical background across all behaviors, whereas severity mediated gender effects for five of the behaviors. Second, to examine the difference in mediation effects across the two countries, we fitted a path model in which all behaviours were jointly predicted by HBM and demographic variables, but we also included regression paths between age/medical background and self-efficacy/benefits, and regression paths between gender and severity. We fitted a path model in which all paths were constrained to be equal across the two samples, except the paths between demographics and HBM variables identified to be different between countries in the results from the model reported in the main manuscript. The model yielded acceptable fit: CFI: .901, RMSEA: .089[.084, .093], SRMR: .064. Third, we used chi-square difference tests to identify specific paths from the demographics to the HBM that should be unconstrained between countries in this overall model. Only the relationship between age and benefits significantly differed between countries and we released this constraint (χ^2^(1) = 5.205, *p* = .023). This model also showed acceptable fit: CFI: .900, RMSEA: .089[.084, .093], SRMR: .063. We show all mediation effects from this overall model in table 3. Important, only the paths from age to benefits were relaxed across the two sites as well as the specific site-variant effects from HBM variables to behaviors (see the main document). Because of these differential constraints, indirect effects reported in ST3 can differ across samples. We report the significant direct effect of demographic variables after mediation in ST4.

| Supplementary Table 2  Significant mediations (indirect effects) of HBM variables between demographics and individual behaviors. | | | | | | |
| --- | --- | --- | --- | --- | --- | --- |
| Behavior | Predictor | Mediator | B | Lower CI | Upper CI | *p* |
| Washing hands | Age | Benefits | .001 | .000 | .001 | .024 |
| Washing hands | Age | Self-Efficacy | .001 | .000 | .003 | .044 |
| Avoid individuals with respiratory infections. | Age | Benefits | .001 | .000 | .001 | .038 |
| Avoid individuals with respiratory infections. | Age | Self-Efficacy | .002 | .000 | .003 | .043 |
| Not touching face. | Age | Benefits | .001 | .000 | .002 | .019 |
| Not touching face. | Age | Self-Efficacy | .002 | .000 | .003 | .046 |
| Covering mouth when sneezing. | Age | Benefits | .001 | .000 | .001 | .032 |
| Covering mouth when sneezing. | Age | Self-Efficacy | .001 | .000 | .002 | .040 |
| Not taking unprescribed medicine. | Age | Self-Efficacy | .001 | .000 | .003 | .045 |
| Disinfecting surfaces. | Age | Benefits | .001 | .000 | .002 | .010 |
| Disinfecting surfaces. | Age | Self-Efficacy | .002 | .000 | .003 | .046 |
| Only using PPE when necessary. | Age | Benefits | .002 | .001 | .003 | .006 |
| Washing hands | Gender | Severity | -.021 | -.035 | -.007 | .004 |
| Avoid individuals with respiratory infections. | Gender | Severity | -.032 | -.050 | -.014 | .000 |
| Avoid individuals with respiratory infections. | Gender | Barriers | -.014 | -.027 | .000 | .043 |
| Not touching face. | Gender | Severity | -.033 | -.054 | -.012 | .002 |
| Disinfecting surfaces. | Gender | Severity | -.045 | -.070 | -.021 | .000 |
| Calling emergency lines when feeling ill. | Gender | Severity | -.046 | -.078 | -.014 | .004 |
| Washing hands | Medical | Benefits | .017 | .002 | .031 | .029 |
| Washing hands | Medical | Self-Efficacy | .066 | .031 | .100 | .000 |
| Avoid individuals with respiratory infections. | Medical | Benefits | .017 | .000 | .033 | .043 |
| Avoid individuals with respiratory infections. | Medical | Self-Efficacy | .077 | .039 | .115 | .000 |
| Not touching face. | Medical | Benefits | .025 | .004 | .046 | .021 |
| Not touching face. | Medical | Self-Efficacy | .081 | .040 | .122 | .000 |
| Covering mouth when sneezing. | Medical | Benefits | .013 | .000 | .026 | .043 |
| Covering mouth when sneezing. | Medical | Self-Efficacy | .049 | .020 | .077 | .001 |
| Not taking unprescribed medicine. | Medical | Self-Efficacy | .070 | .032 | .108 | .000 |
| Disinfecting surfaces. | Medical | Benefits | .030 | .006 | .055 | .014 |
| Disinfecting surfaces. | Medical | Self-Efficacy | .076 | .036 | .115 | .000 |
| Only using PPE when necessary. | Medical | Benefits | .044 | .012 | .075 | .007 |
| Only using PPE when necessary. | Medical | Self-Efficacy | .069 | .030 | .109 | .001 |
| Calling emergency lines when feeling ill. | Medical | Self-Efficacy | .057 | .020 | .095 | .002 |
| *Notes.* Separate models were run for each behavior including all demographic variables and all HBM variables as mediators, the full model outputs can be found on the OSF, Gender was coded as 1 Male 0 Female, Medical was coded as 1 Yes 0 No | | | | | | |
