# Supplementary material for: Testing the Effectiveness of the Health Belief Model in Predicting Preventive Behavior During the COVID-19 Pandemic: The Case of Romania and Italy
Source: Front Psychol. 2022 Jan 12;12:627575. doi: 10.3389/fpsyg.2021.627575 (PMC8789680; doi:10.3389/fpsyg.2021.627575)
Supplement: Supplementary file 3 [file Table_3.docx]

| Suplementary Table 3:  Meditations of demographics by HBM variables (full model) | | | | | | | |
| --- | --- | --- | --- | --- | --- | --- | --- |
| Behavior (outcome) | Demographic predictor | HBM mediator | Country/  Constrained | B | p | Lower CI | Upper CI |
| Washing hands | Gender | Severity | constrained | -.018 | .005 | -.031 | -.006 |
| Avoid individuals with respiratory infections. | Gender | Severity | constrained | -.031 | .000 | -.048 | -.014 |
| Not touching face | Gender | Severity | constrained | -.031 | .001 | -.050 | -.012 |
| Covering mouth when sneezing | Gender | Severity | constrained | -.009 | .083 | -.019 | .001 |
| Not taking unprescribed medicine | Gender | Severity | constrained | .005 | .582 | -.013 | .023 |
| Disinfecting surfaces | Gender | Severity | constrained | -.047 | .000 | -.069 | -.024 |
| Only using PPE when necessary | Gender | Severity | constrained | -.012 | .312 | -.036 | .012 |
| Calling emergency lines | Gender | Severity | constrained | -.042 | .003 | -.069 | -.014 |
| Washing hands | Age | Benefits | Romania | .001 | .017 | .000 | .001 |
| Avoid individuals with respiratory infections. | Age | Benefits | Romania | .001 | .009 | .000 | .001 |
| Not touching face | Age | Benefits | Romania | .001 | .008 | .000 | .002 |
| Covering mouth when sneezing | Age | Benefits | Romania | .000 | .036 | .000 | .001 |
| Not taking unprescribed medicine | Age | Benefits | Romania | .001 | .031 | .000 | .001 |
| Disinfecting surfaces | Age | Benefits | Romania | .001 | .003 | .000 | .002 |
| Only using PPE when necessary | Age | Benefits | Romania | .002 | .001 | .001 | .003 |
| Calling emergency lines | Age | Benefits | Romania | .001 | .009 | .000 | .002 |
| Covering mouth when sneezing | Medical Background | Benefits | Romania | .013 | .050 | .000 | .025 |
| Disinfecting surfaces | Medical Background | Benefits | Romania | .040 | .009 | .010 | .070 |
| Only using PPE when necessary | Medical Background | Benefits | Romania | .061 | .004 | .019 | .102 |
| Covering mouth when sneezing | Age | Self-Efficacy | Romania | .001 | .014 | .000 | .001 |
| Disinfecting surfaces | Age | Self-Efficacy | Romania | .001 | .012 | .000 | .002 |
| Covering mouth when sneezing | Medical Background | Self-Efficacy | Romania | .046 | .001 | .018 | .074 |
| Disinfecting surfaces | Medical Background | Self-Efficacy | Romania | .083 | .000 | .041 | .125 |
| Washing hands | Age | Benefits | Italy | .000 | .987 | .000 | .000 |
| Avoid individuals with respiratory infections. | Age | Benefits | Italy | .000 | .987 | -.001 | .001 |
| Not touching face | Age | Benefits | Italy | .000 | .987 | -.001 | .001 |
| Covering mouth when sneezing | Age | Benefits | Italy | .000 | .987 | -.001 | .001 |
| Not taking unprescribed medicine | Age | Benefits | Italy | .000 | .987 | .000 | .000 |
| Disinfecting surfaces | Age | Benefits | Italy | .000 | .987 | .000 | .000 |
| Only using PPE when necessary | Age | Benefits | Italy | .000 | .987 | .000 | .000 |
| Calling emergency lines | Age | Benefits | Italy | .000 | .987 | -.001 | .001 |
| Covering mouth when sneezing | Medical Background | Benefits | Italy | .026 | .018 | .005 | .048 |
| Disinfecting surfaces | Medical Background | Benefits | Italy | .010 | .220 | -.006 | .027 |
| Only using PPE when necessary | Medical Background | Benefits | Italy | .018 | .096 | -.003 | .040 |
| Covering mouth when sneezing | Age | Self-Efficacy | Italy | .001 | .011 | .000 | .002 |
| Disinfecting surfaces | Age | Self-Efficacy | Italy | .001 | .014 | .000 | .002 |
| Covering mouth when sneezing | Medical Background | Self-Efficacy | Italy | .071 | .000 | .035 | .107 |
| Disinfecting surfaces | Medical Background | Self-Efficacy | Italy | .060 | .001 | .026 | .095 |
| Washing hands | Medical Background | Benefits | constrained | .016 | .027 | .002 | .031 |
| Not touching face | Medical Background | Benefits | constrained | .021 | .017 | .004 | .039 |
| Covering mouth when sneezing | Medical Background | Benefits | constrained | .026 | .013 | .005 | .046 |
| Not taking unprescribed medicine | Medical Background | Benefits | constrained | .018 | .044 | .000 | .035 |
| Calling emergency lines | Medical Background | Benefits | constrained | .027 | .017 | .005 | .050 |
| Washing hands | Age | Self-Efficacy | constrained | .001 | .010 | .000 | .002 |
| Avoid individuals with respiratory infections. | Age | Self-Efficacy | constrained | .001 | .009 | .000 | .002 |
| Not touching face | Age | Self-Efficacy | constrained | .001 | .010 | .000 | .002 |
| Not taking unprescribed medicine | Age | Self-Efficacy | constrained | .001 | .010 | .000 | .002 |
| Only using PPE when necessary | Age | Self-Efficacy | constrained | .001 | .012 | .000 | .002 |
| Calling emergency lines | Age | Self-Efficacy | constrained | .001 | .016 | .000 | .001 |
| Washing hands | Medical Background | Self-Efficacy | constrained | .069 | .000 | .034 | .103 |
| Avoid individuals with respiratory infections. | Medical Background | Self-Efficacy | constrained | .077 | .000 | .041 | .113 |
| Not touching face | Medical Background | Self-Efficacy | constrained | .080 | .000 | .042 | .119 |
| Not taking unprescribed medicine | Medical Background | Self-Efficacy | constrained | .070 | .000 | .035 | .106 |
| Only using PPE when necessary | Medical Background | Self-Efficacy | constrained | .070 | .000 | .033 | .106 |
| Calling emergency lines | Medical Background | Self-Efficacy | constrained | .054 | .001 | .021 | .087 |
| Notes. Gender was coded as 1 Male 0 Female, Medical was coded as 1 Yes 0 No | | | | | | | |
|  | | | | | | | |
