# Supplementary material for: Testing the Effectiveness of the Health Belief Model in Predicting Preventive Behavior During the COVID-19 Pandemic: The Case of Romania and Italy
Source: Front Psychol. 2022 Jan 12;12:627575. doi: 10.3389/fpsyg.2021.627575 (PMC8789680; doi:10.3389/fpsyg.2021.627575)
Supplement: Supplementary file 4 [file Table_4.docx]

| Supplementary Table 4.  Significant paths between demographics and behavior after including mediation by HMB variables. | | | | | |
| --- | --- | --- | --- | --- | --- |
| Behavior | Predictor | B | p | Upper CI | Lower CI |
| Calling emergency lines when feeling ill. | Gender | -.159 | .049 | -.317 | -.001 |
| Disinfecting surfaces. | Gender | -.214 | .001 | -.341 | -.087 |
| Disinfecting surfaces. | Partner | .154 | .024 | .020 | .288 |
| Not taking unprescribed medicine. | Gender | -.250 | .000 | -.383 | -.117 |
| Not taking unprescribed medicine. | Partner | .159 | .021 | .024 | .294 |
| Covering mouth when sneezing. | Gender | -.106 | .020 | -.194 | -.017 |
| Not touching face. | Gender | -.213 | .001 | -.339 | -.088 |
| Avoid individuals with respiratory infections. | Medical Background | -.306 | .000 | -.468 | -.144 |
| Washing hands. | Gender | -.128 | .006 | -.220 | -.037 |
| *Notes.* All paths were constrained to be equal between Romania and Italy, Gender coded as male = 1, Partner coded as yes = 1, Medical Background coded as yes = 1. | | | | | |
