# Supplementary material for: Testing the Effectiveness of the Health Belief Model in Predicting Preventive Behavior During the COVID-19 Pandemic: The Case of Romania and Italy
Source: Front Psychol. 2022 Jan 12;12:627575. doi: 10.3389/fpsyg.2021.627575 (PMC8789680; doi:10.3389/fpsyg.2021.627575)
Supplement: Supplementary file 5 [file Table_5.docx]

**Appendix**

**The health beliefs scale and the recommended preventive behaviors**

| **Variable/Dimension** | **Abbreviation** | **Question** |
| --- | --- | --- |
| **Health beliefs:** to what extent do you agree with the following? (1 – completely disagree; 7 – completely agree) | | |
| Perceived susceptibility to the illness* | SS2 | I have a feeling that I will get infected with Covid-19 |
|  | SS3 | There is a high chance for one to get infected with Covid-19 |
|  | SS4 | There is a high chance for me to get infected with Covid-19 |
|  | SS5 | I feel that I have higher chances to get infected, compared to other people |
| Perceived severity of the illness | SEV1 | The thought of getting infected with Covid-19 scares me |
|  | SEV2 | When I think about Covid-19 my heart beats faster |
|  | SEV3 | I am afraid to think about Covid-19 |
|  | SEV4 | Problems I would experience as a result of an infection with Covid-19 would last a long time |
|  | SEV5 | Getting sick from Covid-19 would threaten a relationship with my boyfriend/girlfriend, teammates, or parents |
|  | SEV6 | Getting sick from Covid-19 would affect my work performance |
|  | SEV7 | If I got sick from Covid-19, my whole life would change |
|  | SEV8 | If I sustained an infection with Covid-19, I would suffer consequences from it for years |
| Perceived benefits of preparing against the illness | BEN1 | When I do Covid-19 prevention actions I feel good about myself |
|  | BEN2 | Adopting prevention recommendations will decrease my risk of getting sick from Covid-19 |
|  | BEN3 | Adopting prevention recommendations will help me with a milder condition, even if I get sick |
| Perceived barriers to preparation against the illness | BAR1 | Other people will consider me weird, if I adopt preventive behaviors against Covid-19 |
|  | BAR2 | Adopting preventive behavior against Covid-19 will be embarrassing to me |
|  | BAR3 | Adopting preventive behavior against Covid-19 will be time consuming |
|  | BAR4 | Adopting preventive behavior against Covid-19 will create physical discomfort |
|  | BAR5 | I don’t have the equipment to adopt preventive behavior against Covid-19 |
| Perceived self-efficacy | SEFF1 | I know how to adopt preventive behavior against Covid-19 |
|  | SEFF2 | I am confident I can adopt preventive behaviors against Covid-19 correctly |
|  | SEFF3 | I have adopted preventive behavior against Covid-19 |
|  | SEFF4 | I would feel confident in adopting preventive behavior against Covid-19 if given the required information |
| **Health behavior:** to what extent you adopted the following behaviors? (1 – completely disagree; 7 – completely agree) | | |
|  | BEHAV1 | Washing hands as often as possible |
|  | BEHAV2 | Avoid contact with other persons suspected of acute respiratory infections |
|  | BEHAV3 | Don't touch eyes, mouth and nose with unwashed hands |
|  | BEHAV4 | Cover your mouth when cough or sneeze |
|  | BEHAV5 | Don't take unprescribed antiviral drugs or antibiotics |
|  | BEHAV6 | Clean surfaces with disinfectants based on chlorine or alcohol |
|  | BEHAV7 | Use protection mask only in case that you suspect you are sick, or provide assistance to sick people |
|  | BEHAV8 | Call emergency if you have fever, or you traveled in countries with confirmed Covid-19 infections |

- The item SS1: “It is very unlikely that I will get infected with Covid-19” was excluded from our study due to differential translations in Romania and Italian
